# ProjectDRIVE: study protocol for a randomized controlled trial to improve driving practices of high-risk teen drivers with a traffic violation

**DOI:** 10.1186/s40621-024-00494-5

**Published:** 2024-03-29

**Authors:** Jingzhen Yang, Corinne Peek-Asa, Ying Zhang, Cara Hamann, Motao Zhu, Yang Wang, Archana Kaur, Robyn Recker, Dominique Rose, Lisa Roth

**Affiliations:** 1 Center for Injury Research and Policy, Abigail Wexner Research Institute at Nationwide Children’s Hospital, 700 Children’s Drive, RB3.5.231, Columbus, Ohio 43205 USA; 2 Department of Pediatrics, The Ohio State University, 700 Children’s Drive, RB3.5.231, Columbus, OH 43205 USA; 3 Office of Research Affairs, University of California at San Diego, San Diego, CA USA; 4 Department of Biostatistics, University of Nebraska Medical Center, Omaha, NE USA; 5 Department of Epidemiology, University of Iowa, Iowa City, IA USA; 6 University of Iowa Injury Prevention Research Center, Iowa City, IA USA; 7 Department of Computer Science and Engineering, The Ohio State University, Columbus, OH USA; 8 Center of Military Psychiatry and Neuroscience, Walter Reed Army Institute of Research, Silver Spring, MD USA

**Keywords:** In-vehicle feedback technology, Parent-teen communication, Driving behaviors, Teen drivers, Traffic violation

## Abstract

**Background:**

Teen drivers with a traffic violation are at increased risk for crashes and crash-related injuries; however, most parent-focused interventions target teen drivers with supervised learner’s permits. Very few interventions are implemented at the probationary driver's license stage or target high-risk teen drivers, such as those with traffic violations. This paper describes the protocol of ProjectDRIVE, A Randomized Controlled Trial to Improve Driving Practices of High-Risk Teen Drivers with a Traffic Violation, which targets improving parent-teen communication about safe driving practices to reduce unsafe driving behaviors and traffic violation recidivism of teen drivers cited for traffic violation.

**Methods:**

Teen drivers (ages 16 or 17) cited for a moving violation and the parent/legal guardian most involved with the teen’s driving are recruited from juvenile traffic courts following their required court hearing. After completing informed consent/assent, enrolled dyads are randomized into one of three groups using stratified block randomization: control, device feedback only, or device feedback plus parent communication training. Participating dyads are followed for 6 months with 3 months of active intervention. Using in-vehicle device and smartphone application technology, the study provides real-time and cumulative driving feedback to intervention teens and collects continually recorded, objectively measured driving outcome data throughout the teen’s study participation. Primary outcomes include rates of risky driving events and unsafe driving behaviors per 1000 miles driven. Secondary outcomes include traffic violation recidivism up to 12 months following study completion and frequency and quality of parent-teen communication about safe driving practices.

**Discussion:**

Through partnership with the local juvenile traffic courts, this study integrates recruitment and randomization into existing court practices. Successfully completing this study will significantly impact juvenile traffic court’s practices and policies by informing judges’ decisions regarding the driving safety programs they refer to teens to prevent motor vehicle crashes and crash-related injuries and deaths.

***Trial registration*** The study was registered on ClinicalTrials.gov Registry (NCT04317664) on March 19, 2020, https://clinicaltrials.gov/study/NCT04317664 and updated on April 27, 2021. This protocol was developed per the SPIRIT (Standard Protocol Items: Recommendations for Interventional Trials) Checklist.

**Supplementary Information:**

The online version contains supplementary material available at 10.1186/s40621-024-00494-5.

## Background

Despite decades of interventions to reduce novice teen driving crashes, motor vehicle collisions remain one of the leading causes of death and disability for teens, particularly for male teens, as well as a high burden for others involved in crashes for which they are responsible (National Center for Injury Prevention and Control 2022; Peek-Asa et al. 2023; Mayhew et al. 2003; McCartt et al. 2003; Insurance Institute for Highway Safety (IIHS) 2021). Teen drivers with traffic violations are at even greater risk for motor vehicle collisions and related hospitalizations and deaths (Factor 2014; Summala et al. 2014). Up to 73% of young adults commit at least one traffic offense within 7 years of receiving their driver’s license (Williams 2003), with a 6-month re-offense rate as high as 56% (Manno et al. 2012; Ekeh et al. 2011). Male teen drivers have the highest rate of recidivism, with an eight to 21 times greater rate of recidivism than females or drivers in other age subgroups (Carnegie et al. 2009). Teen drivers with traffic violations are an understudied and high-risk group that provides an opportunity for tailored interventions (Baird et al. 2013). Parental engagement is vital to educating, supervising, and reinforcing their teens' safe driving behaviors and practices (Curry et al. 2015; Simons-Morton 2007; Peek-Asa et al. 2019 Oct; Mirman et al. 2014; Ramirez et al. 2013). However, as teens start driving without supervision, which is as early as age 16 according to the Ohio licensing and graduated driving laws, their parents are less involved, though teens are continuing to gain driving experience and improve their driving skills (Curry et al. 2015; Simons-Morton 2007). Most existing parent-focused interventions target teen drivers during the supervised learner phase and are implemented as universal interventions for teens of all risk profiles (Curry et al. 2015; Zakrajsek et al. 2013; Winston et al. 2015; Peek-Asa et al. 2014). Few interventions have used selected prevention strategies directed at high-risk drivers under 18, such as those with a traffic violation (Manno et al. 2012; Baird et al. 2013; Mattox 2000; Nirenberg et al. 2013; Watson et al. 2020 Sep), with mixed results in reducing recidivism (subsequent traffic violations) (Mattox 2000). An intervention enrolling high-risk teens and implementing “tiered risk strategies” (Winston et al. 2016) beyond universal parental engagement programs is critical in developing comprehensive teen driving safety programs.

Advances in technology, ranging from in-vehicle devices to smartphone applications, can enhance teens’ safe driving practices by providing real-time, direct feedback to teens and summary reports on teens’ driving behaviors to teens and parents (McCartt et al. 2010; Farmer et al. 2010; Carney et al. 2010; Simons-Morton et al. 2013). However, this technology is rarely used in research to actively engage parents to influence teens’ safe driving practices. The period following a traffic violation presents a unique window for parent engagement (Franklin County Court of Common Pleas 2023). Thus, developing and testing an intervention that simultaneously utilizes in-vehicle driving feedback technology and parent training to influence safe driving practices of high-risk teen drivers with traffic violations could help fill current research and prevention gaps (Peek-Asa et al. 2019 Oct; Alver et al. 2014).

Social Cognitive Theory (SCT) defines human behavior as a triadic, dynamic, and reciprocal interaction of personal factors, behavior, and environmental influences (Bandura 1986). SCT suggests individual determinants (e.g., safe driving skills, attitudes) and the physical and social environment (e.g., in-vehicle driving feedback, parents) influence teens’ safe driving behaviors. Motivational Interviewing (MI) is a “client-centered,” non-confrontational supportive communication strategy. An intervention informed by SCT, evidence-based interventions on parental communication (Yang et al. 2013), and MI principles can train parents to use MI techniques to improve communication. This approach can help enhance parent-teen communications following a teen’s traffic citation and subsequently improve the teen’s safe driving practices (Baird et al. 2013; Nirenberg et al. 2013; Resnicow et al. 2001; Berg-Smith et al. 1999; Miller and Rollnick 2002; Hamann et al. 2019). MI may be particularly effective for teens because it does not directly instruct them what to do but enables them to identify their motivations for adopting safe driving practices (Peek-Asa et al. 2019; Ramirez et al. 2013).

## Objectives

The objectives of ProjectDRIVE, A Randomized Controlled Trial to Improve Driving Practices of High-Risk Teen Drivers with a Traffic Violation (ClinicalTrials.gov: NCT04317664), are to assess how providing driving feedback, with and without training the parent (1) reduces risky driving events and unsafe driving behaviors; (2) increases the frequency and quality of parent-teen communication about safe driving practices; and (3) decreases traffic violation recidivism of teen drivers with a traffic violation. The central hypothesis is that receiving driving feedback via an in-vehicle technology device and smartphone app will reduce risky driving events and behaviors and that augmenting feedback by training parents will further improve intervention outcomes.

## Methods

### Study design and setting

**Fig. 1:**
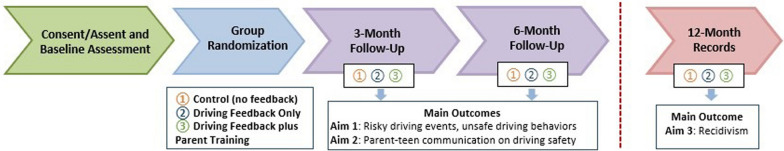
Overview of study design

ProjectDRIVE is a three-group parallel, randomized controlled trial (RCT) with one control and two intervention groups (Fig. 1). The target population is teen drivers (ages 16 or 17) cited for a moving violation (e.g., speeding, failure to obey a traffic signal) and the parent/legal guardian (henceforth ‘parent’) who is most involved with the teen’s driving. According to Ohio law, when a driver under 18 receives a traffic citation, the teen must appear in juvenile traffic court accompanied by a parent to confess or deny committing the violation (Franklin County Court of Common Pleas 2023). This incident provides an ideal opportunity to recruit potential parent-teen dyads. Following their required court hearing, eligible dyads are recruited from county juvenile traffic courts in Ohio (e.g., Franklin, Delaware, Cuyahoga, Summit). After completing informed consent/assent, enrolled dyads are randomly assigned into one of three groups using stratified block randomization:

#### Group 1: control (device and app installation only with no feedback)

The Azūga™ in-vehicle driving feedback technology, consisting of a pager-sized device plugged into the vehicle’s on-board diagnostic (OBD) port in the teen’s car and a smartphone app on the teen’s smartphone, are installed and downloaded, with feedback features disabled (Azūga 2016). Control dyads receive no driving feedback, and parents receive no communication training.

#### Group 2: driving feedback only

The Azūga™ in-vehicle driving feedback device and app are installed as described in Group 1, with the feedback features enabled. Four types of feedback are provided to teens: (1) direct audio feedback; (2) push notification (message on the phone screen when the trip ends); (3) detailed cumulative driving data; and (4) customized bi-weekly driving summary report. Parents do not receive access to the teen’s cumulative driving data, bi-weekly summary report, or communication training.

#### Group 3: driving feedback plus parent training

The Azūga™ in-vehicle driving feedback device and app are installed and used as described in Group 2. In addition, parents receive (1) access to the teen’s cumulative driving data and bi-weekly summary reports, and (2) individualized virtual training in communication strategies about driving safety and a booster session delivered by a traffic safety communication specialist, along with an online parent-teen safe driving communication guide (Peek-Asa et al. 2019; Ramirez et al. 2013; Peek-Asa et al. 2014; Hamann et al. 2019).

**Fig. 2:**
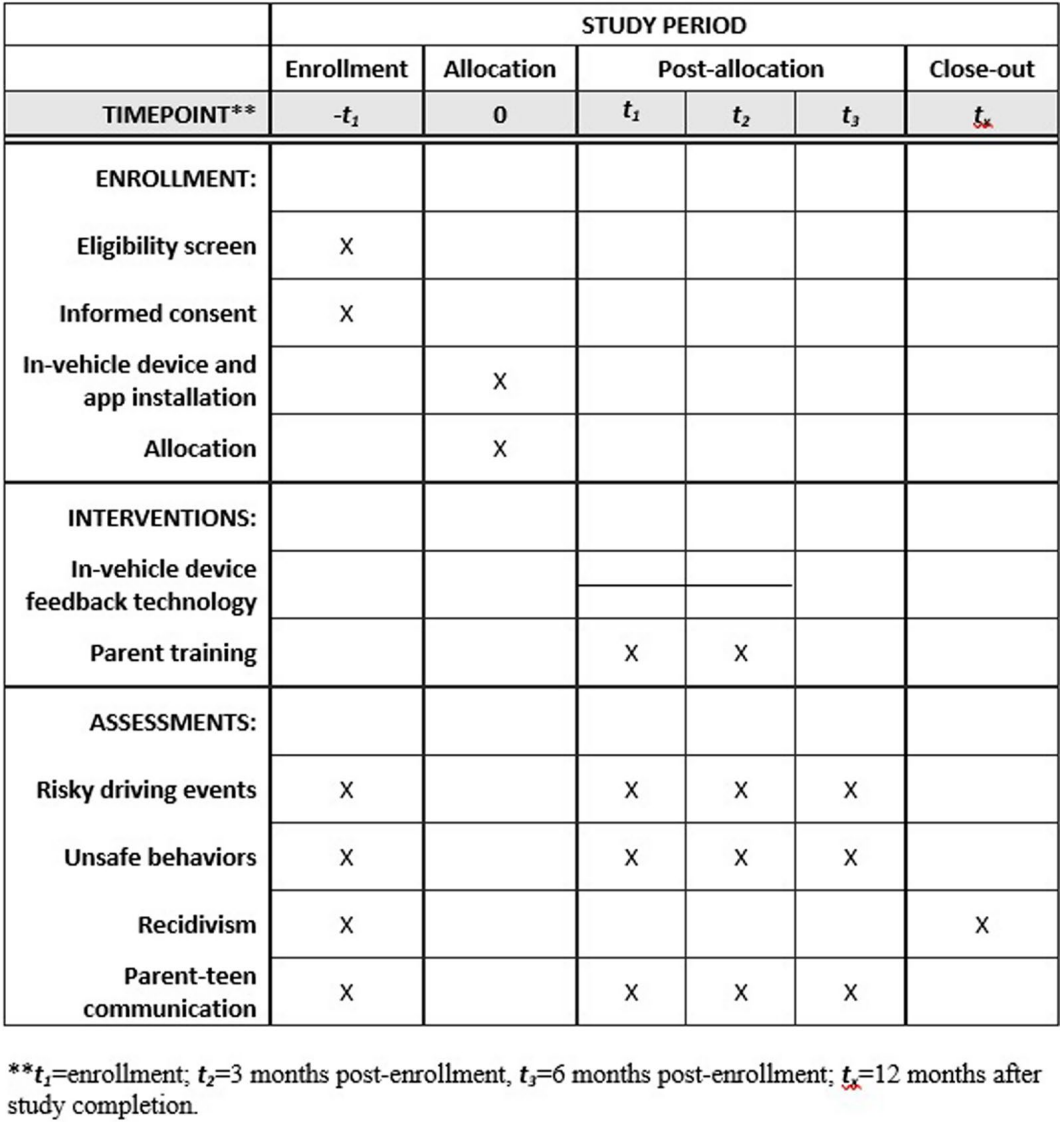
Schedule of enrollment, interventions, and assessments

This report follows SPIRIT (Standard Protocol Items: Recommendations for Interventional Trials) guidelines for reporting a clinical trial protocol (Chan et al. 2013). The schedule of enrollment, interventions, and assessments for ProjectDRIVE is presented in Fig. 2 using SPIRIT-recommended content (Additional file 1).

### Study population and eligibility criteria

ProjectDRIVE aims to enroll 240 parent-teen dyads (80 per group), each including a teen (16 or 17 years) with a moving violation and their parent. Participants are selected without regard to sex, race/ethnicity, or socioeconomic status. Inclusion criteria are (1) age 16–17 at the time of the violation; (2) cited for a moving-related traffic violation; and (3) possess a valid intermediate driver’s license and a Smartphone. Exclusion criteria include (1) inability to drive due to injury, license suspension, or car damage; (2) prior installation of an in-vehicle feedback system; and (3) inactive driving (e.g., driving < 1 h per week).

### Study procedures

#### Recruitment and consent

Research staff recruit at juvenile traffic courts by posting the study flyer at courthouses and distributing the study information sheet to parent-teen dyads at their scheduled court appearance. On recruitment days, research staff briefly introduce the study to potential teens and parents during the judge’s or magistrate’s opening remarks, emphasizing that participation is voluntary and does not impact the court ruling. The judges are also blinded from the identity of the dyads choosing to participate in the study.

After their hearing, interested dyads meet our on-site research staff to learn study details, ask questions, be screened for eligibility, and schedule a follow-up virtual meeting. During the meeting, the research staff obtain consent/assent documents and a photograph of the teen’s driver’s license. The dyad is randomly assigned into one of three study groups. Dyad assignment occurs after a baseline assessment via REDCap (Research Electronic Data Capture), an online secured data collection system. Only dyads with parent and teen consent/assent are enrolled, which may not represent those who decline to participate.

#### Randomization

We stratified our random-block assignment by sex (male vs. female as indicated on the driver’s license) to account for sex differences in traffic violation risk. Before starting the study, 80 blocks of three numbers {1, 2, 3} were randomly generated using *SAS* software. Three study teens, matched by sex, form a block. Group assignment, within each block defined by block ID, strictly follows the randomly generated number order.

#### Data collection

Enrolled dyads are scheduled for study device installation within 2 weeks of enrollment, either in-person at a place and time convenient for participants (e.g., school, library, home) or virtually after the participants receive the device via mail. The researcher installs or guides the teen to install the Azūga™ in-vehicle device in the teen’s car and the Azūga™ app on the teen’s smartphone. The researcher then shows participants how to use the device and app, answers any questions, and provides contact information for questions. Additionally, the research staff schedules parents in Group 3 (Driving Feedback plus Parent Training) for an individualized communication training session with the traffic safety communication specialist and a booster session approximately 2 weeks and 2 months after enrollment, respectively. Research staff regularly monitors the driving data collected via the researchers’ web interface and contacts teens and parents with any problems.

For each dyad, study participation, including the Azūga™ in-vehicle driving data recording, lasts 6 months, with 3 months of active intervention and 3 months of follow-up data collection without intervention. All participating parents and teens complete baseline, three-, and 6-month follow-up surveys online via REDCap asking about the frequency and quality of parent-teen driving safety communications. For each assessment, parents in all three groups are asked to upload a 3-min recorded parent-teen conversation about teen driving. Additionally, teens complete bi-weekly surveys about their risky driving behaviors, including distracted driving behaviors. Parents in Group 3 and traffic safety communication specialists complete an online survey following each virtual communication training session. Court records are obtained via a special data request to measure recidivism retrospectively up to 12 months after the study’s completion.

## Two intervention components

### In-vehicle device feedback technology

**Fig. 3:**
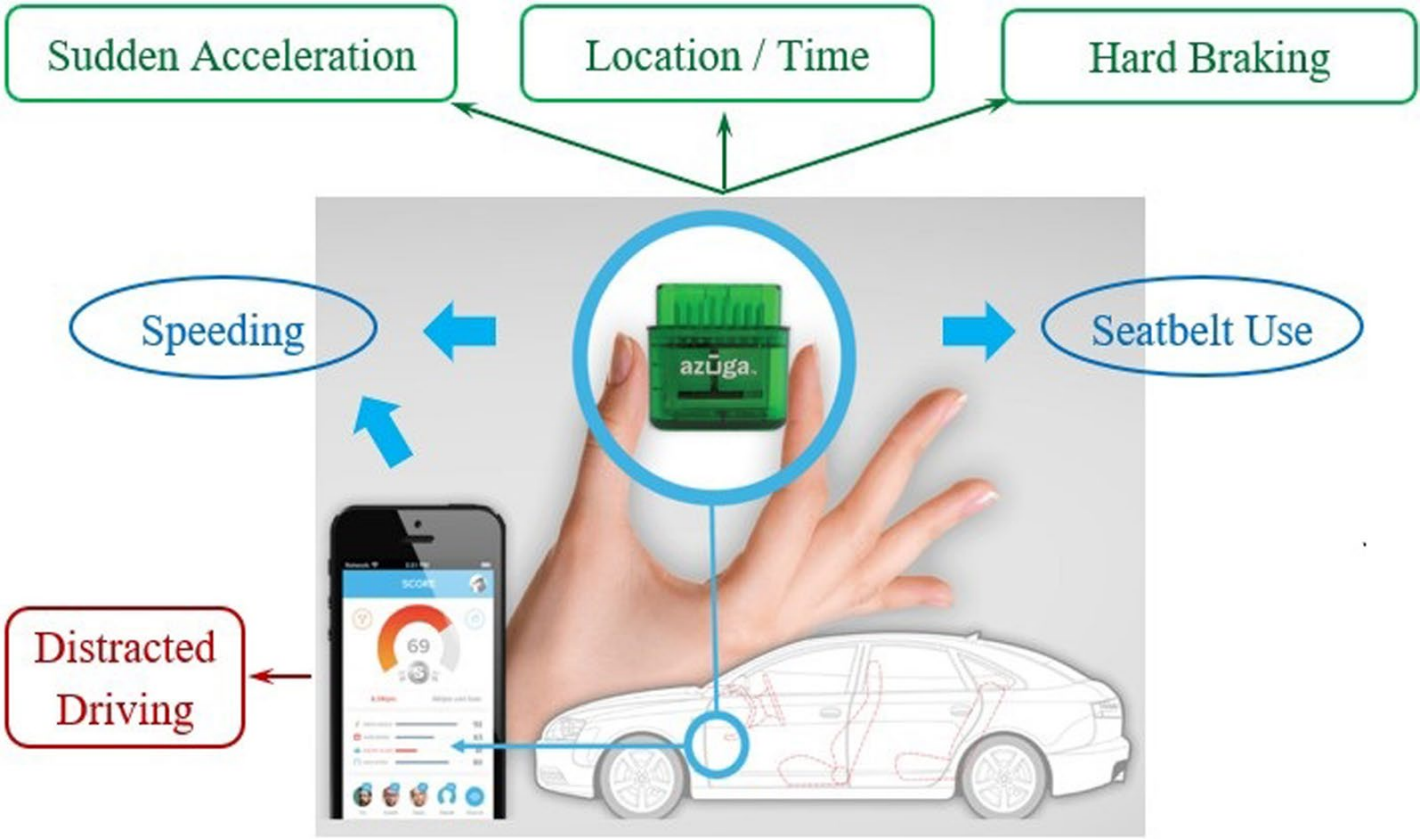
In-vehicle driving feedback technology

Teens (Groups 2 and 3) receive real-time and cumulative feedback on the teen’s driving via the Azūga™ in-vehicle device and smartphone app (Fig. 3) (Azūga 2016). Parents (Group 3) receive cumulative feedback only. The Azūga™ in-vehicle device is not visible while driving and does not interfere with vehicle features; it measures trip routes (via GPS trace), risky driving events (speeding, hard braking, sudden acceleration), and unsafe behaviors (speeding duration, no seatbelt use). The pager-size device, installed in seconds (Azūga 2016), plugs into the vehicle’s OBD port (available in all cars manufactured after 1996). As part of the Azūga™ package, the Azūga™ Smartphone App pairs with the participating teen’s smartphone via Bluetooth using a unique username and password to detect seatbelt use in most vehicle models and speeding duration. When the device is not in cellular connectivity, the OBD device plugged into the vehicle captures and stores the driving data and uploads the data to the account once the network returns (Azūga 2016).

The Azūga™ in-vehicle device and app provide four types of driving feedback by source and recipient(s) (Table 1). Real-time feedback occurs through audio beeps when a g-force event is triggered. Cumulative driving data are available through the participant’s web interface and the smartphone app. All recorded data collected via the in-vehicle driving feedback app are transmitted automatically via cellular modems to a secure cloud server designated for our study (Azūga 2016). The research team processes and programs the customized, real-time driving data before automatically uploading it to a secured Nationwide Children’s Hospital’s “Researcher” web interface available only to approved research personnel. The research team links the data to study participants via their unique username (e.g., email address) and delivers cumulative driving data and feedback via the participant’s web interface, the Azūga™ smartphone app, email, and text message. The role of the tech company in the study includes providing in-vehicle device technology, driving data, and technical support as needed.

**Table 1:** Type of feedback from Azūga™ in-vehicle technology and app, by time, source, and recipient

Type of feedback	Time of feedback	Source of feedback	Description	Recipient
1. Repeated beeps	Real time	In-vehicle device	Feedback is triggered by risky driving events when the vehicle movement exceeds a set threshold of gravitational force (g-force) (hard braking ≤ − 0.45 g-force; sudden acceleration > 0.35 g-force).	Teens in Groups 2&3
2. Push notification (message on the phone screen)	Real time when a trip ends	In-vehicle device and app	Feedback is provided via a push notification when (1) driving speed > 10 miles per hour over the posted speed limit, based on Google Maps™ Roads API and OpenStreetMap; and (2) the driver does not buckle their seatbelt for certain models and years. Teens can review at the end of each drive.	Teens in Groups 2&3
3. Cumulative driving data	Anytime	In-vehicle device and app	Data can be viewed retrospectively, including trip routes, location, and time of risky driving event(s) and unsafe behavior(s).	Teens in Groups 2&3, Parents in Group 3
4. Bi-weekly driving summary report	Bi-weekly	Research team	Customized reports are generated and include 1) a summary of trips, risky driving events, and unsafe behaviors, including the teen’s score compared with all study participants; and 2) feedback on safe driving behavior(s) with suggestions to mitigate the unsafe behavior(s).	Teens in Groups 2&3, Parents in Group 3

### Parent training

Parents in Group 3 receive guidance and communication strategies to augment the in-vehicle driving feedback and to enable parents to effectively communicate with their teens about specific safe driving topics (e.g., speeding, using a seatbelt, maintaining safe distance) based on objectively recorded driving data. Training is based on the program Steering Teens Safe, developed by our team (Peek-Asa et al. 2019; Peek-Asa et al. 2014; Hamann et al. 2019). This evidence-based intervention aims to improve safe teen driving by enhancing parental communication skills to significantly reduce risky driving behaviors among teen drivers (Peek-Asa et al. 2019; Peek-Asa et al. 2014; Hamann et al. 2019).

Our parent training includes two activities:

Individualized virtual communication training and booster with a traffic safety specialist

Training is provided virtually to parents via Zoom or Microsoft Teams within 2 weeks of enrollment. A 50-min initial training provided by an experienced traffic safety communication specialist focuses on specific skills derived from MI for effective parent-teen communication. MI techniques include “OARS” (i.e., Open-ended questions, Affirmations, Reflective listening, and Summarizing) (Resnicow et al. 2001; Berg-Smith et al. 1999; Miller and Rollnick 2002). Parents are trained to begin communication by soliciting input from their teens, focusing on objective behaviors and expressing emotional responses, and using active listening to explore and support self-motivation for safe driving. Parents learn the “rolling with resistance” technique, which explores and helps overcome barriers to safe driving practices. Parents practice these strategies and skills using role-play during the virtual training session with the specialist.

Following the individualized virtual training, parents receive bi-weekly email reminders to review their teen’s driving data from the in-vehicle technologies, practice learned communication strategies with their teen, and complete post-intervention surveys (including submitting a voice-recorded [approximately 3 min] parent-teen conversation to be used for the tailored booster session). The communication specialist reviews and discusses the data with parents during the individualized virtual booster session 2 months after enrollment, reinforcing communication strategies and techniques and measuring the parent’s success in using them.

An online parent-teen safe driving communication guide

This online guide, designed for parents of this study, is provided to parents after the individualized virtual communication training and supports the learned skills and specific talking points about safe driving. The online guide includes three MI demonstration videos and 26 safe driving lessons. The three videos (each under 3 min) have examples of MI-style parent-teen conversations involving seat belt use, distracted driving, and safe car maneuvering. The 26 safe driving lessons include general topics such as basic safety principles (e.g., seat belt use and distracted driving), important driving skills (e.g., maintaining a safe distance), special driving situations (e.g., bad weather), and setting restrictions for driving (e.g., no texting while driving) as well as specific topics based on the feedback received from the Azūga™ app. The content of each lesson is customized to our study participants and includes the rationale and importance of the topic, talking points, and conversation starters for effective parent-teen conversations. The conversation starters are designed with written text and audio illustrations of typical vs. MI conversation starters utilizing OARS techniques. Parents are instructed to complete all 26 safe driving lessons during the 3-month active intervention period, with two to three lessons assigned per week. Prior to the parent-teen conversations, parents are advised to review and complete lessons based on their teen’s bi-weekly driving summary report.

### Strategies to improve intervention fidelity

To ensure the fidelity of the driving feedback intervention, research staff monitor the “Researcher” web interface daily to confirm the in-vehicle device is collecting driving data and/or providing feedback properly and following an established protocol to solve any device-related technical or other issues.

To ensure the fidelity of the parent training, this study uses an established intervention script and materials. An experienced communication specialist delivers the training. Research staff record and review the training and booster sessions, conduct participant and trainer surveys immediately after each intervention using the Behavior Change Counseling Index (Lane et al. 2005), and keep participation logs.

Research staff track the participants’ engagement with intervention components using Google Analytics and remind participants via bi-weekly emails and text messages to review the driving data and summary report.

### Study measures (summarized in Table 2)

**Table 2:** Main outcome measures organized by domain, data source, and assessment time

Domain	Measure	Data source	Time point				
			BL	1–2	3	6	12
Outcome variables							
Risky driving events	Hard braking^a^	Technology		×	×	×	
	Sudden acceleration^a^	Technology		×	×	×	
Unsafe driving behaviors	Speeding^a^	Technology		×	×	×	
	Distracted driving^b^	Survey^T^		×	×	×	
	No seatbelt use^a,b^	Technology		×	×	×	
Recidivism	No. of citations (in 12 months)^a^	Court Data					×
	Days from initial to next citation^a^	Court Data					×
Parent-teen communication	Frequency	Survey^T,P^	×	×	×	×	
	Quality	Survey^T,P^	×	×	×	×	
	Voice-recorded conversation	Recorder^P^		×	×	×	
Intervention implementation	Engagement with feedback	Web Tracking^a,c,d^		×	×	×	
	Engagement with training	Web Tracking^a,d^/Survey^d^		×	×	×	
	Evaluation of training delivery	Survey^d^		×			
Other variables	Demographic characteristics	Survey^T,P^	×				
	Driving habits questionnaire	Survey^T^			×	×	
	Parental communication patterns	Survey^T,P^	×		×	×	

*BL* baseline, *T* teens, *P* parents

^a^Continuous data collection

^b^Bi-weekly survey

^c^Teens in Groups 2 and 3

^d^Parents in Group 3 only

#### Primary outcome

*Risky driving events* (Table 2) are collected continuously via the Azūga™ in-vehicle device and the smartphone app for 6 months for teens in all three groups. The number and type of driving events, including speeding (> 10 miles per hour over the posted speed limit), hard braking (≤ − 0.45 g-force), and sudden acceleration (> 0.35 g-force), are automatically coded and counted in the system. The rates are computed as the number of risky driving events divided by miles driven and multiplied by 1,000.

*Unsafe behaviors* (Table 2) are collected continuously via the Azūga™ in-vehicle device and the smartphone app for 6 months for teens in all three groups. The type of driving behaviors (e.g., speeding and no seatbelt use) and the duration (e.g., miles driven) are automatically coded and counted in the Azūga™ system. The unsafe behavior proportions are calculated as miles driven with an unsafe behavior divided by total miles driven, then multiplied by 1000 (e.g., proportion of 1000 miles without wearing a seatbelt). Additionally, bi-weekly surveys will collect self-reported distracted driving (e.g., calls made and received, texts sent and viewed while driving, searches for a webpage/app) and seatbelt use (since not all vehicle models are available via Azūga™ in-vehicle device).

#### Secondary outcomes

*Recidivism* (Table 2) is measured in all three groups by linking traffic citation(s) and court disposition data obtained by special request using the participating teen’s driver’s license number. Recidivism data are collected and analyzed up to 12 months after study completion, including the date, type of violation, and days from index violation to subsequent violation.

*Parent-teen communication* (Table 2) is measured among teens and parents in all three groups at baseline and at 3- and 6-months (via REDCap), using the survey instrument adopted from our prior studies (Hamann et al. 2019; Harland et al. 2021). Dyads are asked to rate the level of success (1 = poor to 10 = excellent) and frequency of parent-teen conversations on each of the 26 common driving skills/safety principles (Harland et al. 2021) discussed in the past month (0 = never to 3 = often). The frequency of parent-teen communication (Table 2) ranges from 0 to 78, with higher scores indicating more frequent communication. Quality of parent-teen communication (Table 2) scores are calculated by averaging ratings for all addressed skills/principles, weighting them based on the maximum score possible, and then recording scores as a percentage (possible range = 1–100%). Additionally, the quality of parent-teen communication is assessed using voice-recorded dyad conversations (one conversation submitted per survey). Two trained coders with established inter-rater reliability code conversations to identify if parents (1) use active listening, (2) use OARS, (3) solicit input about the teen’s perspective, (4) focus on objective behaviors, and/or 5) express emotional responses, with each item scored individually (0 = never to 3 = often) (Berg-Smith et al. 1999; Miller and Rollnick 2002). A summary score for each conversation is calculated.

#### Intervention implementation process variables (Table 2)

*Engagement with device feedback* is measured among teens in Groups 2 and 3 and parents in Group 3 via online tracking of the participant’s web interface using Google Analytics. The number of times each driving summary is accessed (links clicked) and the time spent at each link is recorded.

*Engagement with communication training* is measured among parents in Group 3 using a self-report questionnaire and online tracking. Following each individualized training, parents report (1) how frequently they have used communication strategies in their parent-teen discussions on safe driving during the past month (0 = never to 3 = often); (2) how helpful these strategies and techniques have been (1 = not helpful at all to 10 = extremely helpful); and (3) their perceived level of mastery of the strategies and techniques (1 = poor to 10 = excellent). Summary scores are calculated. Using Google Analytics, parents’ interactions with the parent-teen safe driving communication guide are tracked, including logins, number of visited sub-links, and time spent at each link.

*Communication training delivery* is evaluated among parents in Group 3 and communication specialist via participant and trainer surveys completed immediately after each training session using the Behavior Change Counseling Index (Lane et al. 2005).

#### Other variables

Other variables collected via self-report from all three groups as potential confounders include dyad demographic information*:* teen’s sex, age, race/ethnicity, driver’s license information (via license photo), and date and type of traffic violation, and parent’s sex, age, race/ethnicity, relationship to the teen, marital status, and education level. Both parent and teen driving habits/crash and violation histories include a history of distracted driving, seatbelt use, and crash-related events, including violations and crashes (either as a driver or passenger). Additional data collected from teens include vehicle make, model, and VIN #; age at first driving experience; age at licensure; any participation in a teen driving safety program(s); hours driven per week; and items from the Driving Habits Questionnaire concerning difficulties/avoidance in specific driving situations (e.g., driving at night, inclement weather) (Owsley et al. 1999).

*Parental communication patterns* are assessed using the Family Communication Pattern Instrument, a 26-item Likert scale (Chaffee et al. 1973; Fitzpatrick and Ritchie 1994; McLeod et al. 1972). The instrument contains two subscales: conformity orientation and conversation orientation (reliability of 0.76 and 0.84, respectively) (Chaffee et al. 1973; Fitzpatrick and Ritchie 1994; McLeod et al. 1972). Based on the two subscale scores, parental communication patterns are coded into four types: (1) pluralistic, (2) protective, (3) consensual, and (4) laissez-faire (Yang et al. 2013; Hamann et al. 2019; Chaffee et al. 1973).

## Analytic plan

### Data management

#### Azūga™ data

All Azūga™ data (collected in 2-min intervals) are transferred automatically daily to a protected research server only accessible to a designated subgroup of study staff. We first use custom-developed software to conduct monthly quality checks of the downloaded Azūga™ data to validate trips, events, and driving activity duration. We then merge Azūga™ data with other participant data collected via REDCap. Finally, we conduct preliminary data analysis at participant and event levels to identify issues before primary and secondary analyses.

#### Missing data

To handle missing data, we will first examine patterns of missing data and identify missing data mechanisms. If the missing data are rare and appear to be independent of study outcomes, we will treat them as missing completely at random and conduct the data analyses ignoring the missing observations. Otherwise, we will use a multiple imputation method with a simulation-based approach from a model that describes the missing mechanism in our primary analyses (Little 1988; Rubin and Schenker 1991).

### Data analysis

#### Descriptive analysis

Data will be analyzed to address our *central hypothesis* that direct feedback via technological devices will reduce risky driving behaviors, and augmenting direct feedback with parent training will further reduce these behaviors. Additionally, we will conduct a process evaluation by assessing engagement with intervention components and fidelity and include these variables as covariates when assessing intervention effects. All analyses will adjust for relevant baseline characteristics and other potential covariates (e.g., teen’s age, race/ethnicity, history of crashes or near-crashes, driving frequency and time, parental communication patterns, etc.), and we will consider adjusting the p-value when multiple outcomes are assessed. We will also examine program effects by sex to determine if the program is differentially effective.

#### Primary analysis

For the primary outcomes of risky driving events (speeding, hard braking, and sudden acceleration), we will calculate the overall rate and rate for each type of event per 1,000 miles driven for each teen at 3- and 6-month follow-ups. One-way ANOVAs along with the post-hoc analysis of pairwise 95% confidence intervals will compare the differences in rates per 1,000 miles among the three groups, adjusting for multiple comparisons. In addition, to model the rates of risky driving events, we will conduct a Quasi-Poisson analysis for over-dispersed count data (Ver Hoef and Voverng 2007), where two dummy variables indicating Groups 2 and 3 will be created as covariates to determine risk ratios of risky driving events among the three groups at 3- and 6-month follow-ups, adjusting for the other potential covariates described above. The p-values will adjust for multiple tests, considering multiple outcome measures are included.

For the primary outcomes of unsafe driving behaviors (speeding duration, distracted driving, and no seatbelt use), we will calculate the total proportion and proportion for each type of behavior per 1000 miles driven during the 3- and 6-months from enrollment for each teen. To model the proportion of unsafe behaviors per 1000 miles driven, we will implement a Quasi-Poisson analysis for over-dispersed count data, where two dummy variables indicating Groups 2 and 3 will be created as covariates to determine the risk ratios of unsafe driving behavior among the three groups at 3- and 6-month follow-ups, adjusting for the potential covariates described above. In addition, we will conduct a secondary analysis of Pearson correlation coefficients to ascertain how risky driving events and unsafe driving behaviors are correlated and whether the correlations are different among groups. Potential covariates will be adjusted as described above.

For the secondary outcome of teen traffic violation recidivism, we will estimate Kaplan–Meier curves of the survival probability of recidivism for the three groups and use a Log-Rank test to compare the differences in risk of recidivism among the three groups (Klein and Moeschberger 2003). We will also use the Cox Proportional Hazards Model to ascertain whether and how much the Device Feedback Only and/or Device Feedback plus Parent Training intervention reduces the hazard of teen recidivism, adjusting for the potential covariates described above. We will define the event of interest as recidivism and the survival time as the number of days from enrollment to recidivism or 12 months after completing the study (censored). In addition, we will specifically include the measures of risky driving and unsafe driving behaviors in the Cox Proportional Hazards Models to examine how risky driving events and unsafe driving behaviors increase the hazards of recidivism, adjusting for potential covariates (Klein and Moeschberger 2003). We will conduct a model diagnosis for the proportional hazards assumption; if it is violated, other survival models, such as an accelerated failure time model, will be explored. The time of the first traffic violation will be included as a covariate to test the effect of the seasonality of the violation.

For the secondary outcome of frequency and quality of parent-teen communication about driving safety, we will calculate mean scores and adjusted mean scores for parents and teens at baseline and 3- and 6-month follow-ups, and the summary scores coded from parent-teen conversations. All three outcome measures will be scaled numerically. To ascertain the differences in frequency and quality of parent-teen communication among the three groups, ANCOVAs will be used for each outcome, adjusting for the potential covariates described above (Gamst et al. 2008).

#### Secondary/exploratory analysis

We will perform the following secondary analyses: (1) correlation analysis to examine if and how frequency and quality of parent-teen communication are correlated; (2) longitudinal data analyses using mixed-effect models to further ascertain if parent training enhances parent-teen communication over time with a focus on how these measures change in Group 3; and (3) further analysis on risky driving events and unsafe driving behaviors using a Quasi-Poisson analysis by including the measures of frequency and quality of parent-teen communication as three additional covariates to evaluate the effects of parent-teen communication, adjusting for group differences.

#### Sample size

The sample size was determined for power based on the primary outcome, the rate of risky driving events collected via our pilot studies, and results from our previous RCTs (Peek-Asa et al. 2019; Peek-Asa et al. 2014). We set a study reduction rate of 50% for Group 2 vs. Group 1 and Group 3 vs. Group 2, respectively. The risky driving events are count data, which are modeled by Quasi-Poisson distribution with an over-dispersion factor of 25 (based on our pilot study, $\frac{Var}{{Mean}} = \frac{{32.7^{2} }}{43} = 24.9$) for the ordinary Poisson variability (Muthén and Muthén 2002). We assume the three groups have the same over-dispersion factor for the Quasi-Poisson distributed data. With these design parameters and three groups of an equal sample size of 74, this study has 80% or higher power at a significance level of 0.05 to detect that Group 2 would have fewer risky driving events than Group 1. Group 3 would have fewer risky driving events than Group 2, using a one-way ANOVA and adjusting for two-sided multiple comparisons (Gamst et al. 2008). Based on these sample sizes and less than 10% loss to follow-up, 80 dyads per group (n = 240) will be recruited.

### Patient and public involvement

The results from the pilot study conducted with teen drivers cited for a moving violation and their parents have informed the study protocol and instruments used. Before the study, the research team also met several times with juvenile court judges or magistrates to solicit their input before developing the recruitment and data collection strategies. Additionally, the research team observed court practices and developed study procedures sanctioned by each of the juvenile court judges and magistrates. Each participating juvenile traffic court has also provided a private room/space for recruitment and randomization. The protocol includes a plan to solicit feedback from participating families at the end of their participation to enhance implementation as needed.

### Ethics

This study has received ethical approval from the Institutional Review Board (IRB) at the participating institutions as a single IRB (Nationwide Children’s Hospital IRB17-00318). The IRB must approve a modification request before implementation if any protocol changes are needed. To ensure individuals are not coerced to participate in the study by the court, all meetings between participants and research staff only occur after the teens’ court hearing, and all judges are blinded from the identity of any teens and parents who choose to participate. A Data and Safety Monitoring Plan has been developed for this trial, including creating a Data and Safety Monitoring Committee comprised of experts in clinical and translational research who are collaborating with the research team throughout the study to ensure safeguards are in place to prevent any adverse events (e.g., crashes or injuries) and to determine whether the observed frequency and type of events exceed those expected in the population. Adverse events will be reported to the required entities per IRB policy, depending on the involvement of risks to subjects or others.

A Certificate of Confidentiality is automatically issued for the participants in this study as part of the NIH funded randomized trial. Before data collection, we took extra steps to develop and implement the data collection, transmission, and storage procedures to ensure that study data are securely maintained and compliant with applicable laws and regulations. We also use the secure Azūga™ Amazon web server and the secure Nationwide Children’s Hospital server to protect all study data, and in particular, data collected from the in-vehicle device (e.g., GPS data). Several precautions have been taken to protect participant confidentiality and privacy, including anonymizing participants (only using Azūga™ participant aliases, rather than real names), isolating data (storing the study data on a physically isolated drive separate from all other Azūga™ clients’ data), controlling access (only trained researchers can access the data), and representing participants by computer-assigned case numbers, instead of names, images, and/or specific identifiers. Further, teens and parents are assured that only trained researchers have access to the data.

### Dissemination

We will use common strategies to disseminate our study findings, including (1) traditional academic outreach (e.g., publications in peer-reviewed journals and presentations at professional conferences); (2) media outreach (e.g., radio, TV, and social media) and creating related materials (e.g., reports, special interest newsletters); (3) key stakeholders and organizations (e.g., juvenile traffic courts, Teen Safe Driving Coalition, National Foundation for Teen Safe Driving). We will disseminate the findings of this study (edited in lay language) to study participants as well as judges and staff in juvenile traffic courts through social media posting and infographics. In addition, we will collaborate with our research center’s Translational Research Team, consisting of experts trained in both injury prevention and communications, who will disseminate our research findings to various audiences through a number of platforms, ensuring that our research will be widely disseminated, and will therefore have the greatest potential to influence policy and practice.

## Discussion

In partnership with the local juvenile traffic courts, ProjectDRIVE integrates recruitment and randomization into existing court practices, a novel project component. Completing this RCT will help determine whether an in-vehicle driving feedback technology plus parent training can improve parent-teen communication and reduce unsafe driving events, behaviors, and recidivism of teen drivers. We will further demonstrate the feasibility of embedding prevention programs effectively into juvenile courts.

The COVID-19 pandemic has added challenges to this trial, including case backlog, delayed traffic citation processing time due to COVID-19 lockdown orders, and changes in traffic court practices to reduce contact. The research team requested and received a mid-year project extension approved by the funder for 12 months (from May 1, 2021, to April 30, 2022). The research team has developed tools to obtain consent, enroll study participants, and conduct all activities virtually. The research team also adjusted eligibility criteria to extend days between the citation and enrollment to accommodate delayed court case processing and implemented social media recruitment to broaden the study reach to increase enrollment.

Numerous novel technological approaches have emerged to improve safe teen driving. However, a paucity of studies has examined how these approaches can be incorporated into targeted interventions designed for teen drivers based on their risk for motor vehicle collisions. As commercially available technological strategies to monitor teen driving practices continue to develop, understanding not only if these strategies work but also how they work is critical. ProjectDRIVE explicitly tests the impact of technological approaches with and without parent training in communication strategies on teen driving practices, which fills a critical gap in the literature. More importantly, the study findings will significantly impact juvenile traffic court practices and policies by informing judges’ decisions regarding the type of driving safety program they refer to teens to prevent motor vehicle collisions and related injuries and deaths.

### Supplementary Information

**Additional file 1.** SPIRIT Checklist.

## Data Availability

The datasets used and/or analyzed during the current study are available from the corresponding author upon reasonable request. The corresponding author will be responsible for providing access to research data requested by third parties as freely and timely as possible unless a legal obligation restricts access to the data (e.g., non-disclosure agreement), intellectual property protection, ethical approval requirements, ethical or security reasons, or other legitimate reasons.

## Abbreviations

IRB: Institutional review board
MI: Motivational interviewing
OARS: Open-ended questions, affirmations, reflective listening, and summarizing
OBD: On-board diagnostic
RCT: Randomized controlled trial
REDCap: Research electronic data capture
SCT: Social cognitive theory

## References

[CR1] Alver Y, Demirel MC, Mutlu MM (2014). Interactions between socio-demographic characteristics: traffic rule violations and traffic crash history for young drivers. Accid Anal Prev.

[CR2] Azūga. Azūga GPS Device. Azūga, Inc. https://azuga.com/technical-specs/. Accessed 24 May 2016.

[CR3] Baird J, Nirenberg TD, Longabaugh R, Mello MJ (2013). The effect of group-adapted motivational interviewing on traffic convictions and driving behaviors of court-adjudicated youth. Traffic Inj Prev.

[CR4] Bandura A (1986). Social foundations of thought and action: a social cognitive theory.

[CR5] Berg-Smith SM, Stevens VJ, Brown KM (1999). A brief motivational intervention to improve dietary adherence in adolescents. The Dietary Intervention Study in Children (DISC) research group. Health Ed Res.

[CR6] Carnegie JA, Strawderman WE, Li W. Study of recidivism rates among drivers administratively sanctioned by the New Jersey motor vehicle commission. No. FHWA-NJ-2009-019. 2009.

[CR7] Carney C, McGehee DV, Lee JD, Reyes ML, Raby M (2010). Using an event-triggered video intervention system to expand the supervised learning of newly licensed adolescent drivers. Am J Public Health.

[CR8] Chaffee SH, McLeod JM, Wackman DB, Dennis J (1973). Family communication patterns and adolescent political participation. Socialization in politics: a reader.

[CR9] Chan A-W, Tetzlaff JM, Gøtzsche PC, Altman DG, Mann H, Berlin J, Dickersin K, Hróbjartsson A, Schulz KF, Parulekar WR, Krleža-Jerić K, Laupacis A, Moher D (2013). SPIRIT 2013 explanation and elaboration: guidance for protocols of clinical trials. BMJ.

[CR10] Curry AE, Peek-Asa C, Hamann CJ, Mirman JH (2015). Effectiveness of parent-focused interventions to increase teen driver safety: a critical review. J Adolesc Health.

[CR11] Ekeh AP, Hamilton SB, D'Souza C, Everrett E, McCarthy MC (2011). Long-term evaluation of a trauma center-based juvenile driving intervention program. J Trauma.

[CR12] Factor R (2014). The effect of traffic tickets on road traffic crashes. Accid Anal Prev.

[CR13] Farmer CM, Kirley BB, McCartt AT (2010). Effects of in-vehicle monitoring on the driving behavior of teenagers. J Saf Res.

[CR14] Fitzpatrick MA, Ritchie LD (1994). Communication schemata within the family: multiple perspectives on family interaction. Hum Commun Res.

[CR15] Franklin County Court of Common Pleas. https://drj.fccourts.org/Rules.aspx?rtype=J&rid=54&F=E. Accessed 4 Dec. 2023.

[CR16] Gamst G, Meyers LS, Guarino AJ (2008). Analysis of variance designs: a conceptual and computational approach with SPSS and SAS.

[CR17] Hamann C, Schwab-Reese L, O'Neal EE, Butcher B, Yang J, Peek-Asa C (2019). Family communication patterns and teen driving intervention effectiveness. Am J Health Behav.

[CR18] Harland KK, Yang JZ, Peek-Asa C (2021). Steering Teens Safe: translation to a workplace wellness program in the United States. Health Promot Int.

[CR19] Insurance Institute for Highway Safety (IIHS). Fatality facts: teenagers 2021. Arlington (VA): The Institute; 2023. https://www.iihs.org/topics/fatality-statistics/detail/teenagers. Accessed 4 Dec 2023.

[CR20] Klein JP, Moeschberger ML (2003). Survival analysis: techniques for censored and truncated data.

[CR21] Lane C, Huws-Thomas M, Hood K (2005). Measuring adaptations of motivational interviewing: the development and validation of the behavior change counseling index (BECCI). Patient Educ Couns.

[CR22] Little RJA (1988). A test of missing completely at random for multivariate data with missing values. J Am Stat Assoc.

[CR23] Manno M, Maranda L, Rook A, Hirschfeld R, Hirsh M (2012). The reality of teenage driving: the results of a driving educational experience for teens in the juvenile court system. J Trauma Acute Care Surg.

[CR24] Mattox J (2000). The impact of a court-directed parental involvement program on parental monitoring and young-driver behaviors.

[CR25] Mayhew DR, Simpson HM, Pak A (2003). Changes in collision rates among novice drivers during the first months of driving. Accid Anal Prev.

[CR26] McCartt AT, Shabanova VI, Leaf WA (2003). Driving experiences, crashes, and teenage beginning drivers. Accid Anal Prev.

[CR27] McCartt AT, Farmer CM, Jenness JW (2010). Perceptions and experiences of participants in a study of in-vehicle monitoring of teenage drivers. Traffic Inj Prev.

[CR28] McLeod JM, Chaffee SH, Tedeschi JT (1972). The construction of social reality. The social influence process.

[CR29] Miller WR, Rollnick S (2002). Motivational interviewing: preparing people for change.

[CR30] Mirman JH, Curry AE, Winston FK (2014). Effect of the teen driving plan on the driving performance of teenagers before licensure. JAMA Peds.

[CR31] Muthén LK, Muthén BO (2002). How to use a Monte Carlo study to decide on sample size and determine power. Struct Equ Model.

[CR32] National Center for Injury Prevention and Control; Centers for Disease Control and Prevention. Teen Driver and Passenger Safety. 2022. https://www.cdc.gov/transportationsafety/teen_drivers/index.html. Accessed 4 Dec 2023.

[CR33] Nirenberg T, Baird J, Longabaugh R, Mello MJ (2013). Motivational counseling reduces future police charges in court referred youth. Accid Anal Prev.

[CR34] Owsley C, Stalvey B, Wells J, Sloane ME (1999). Older drivers and cataract: driving habits and crash risk. J Gerontol A Biol Sci Med Sci.

[CR35] Peek-Asa C, Cavanaugh JE, Yang JZ, Chande V, Young T, Ramirez M (2014). Steering teens safe: a randomized trial of a parent-based intervention to improve safe teen driving. BMC Public Health.

[CR36] Peek-Asa C, Reyes ML, Hamann CJ, Butcher BD, Cavanaugh JE (2019). A randomized trial to test the impact of parent communication on improving in-vehicle feedback systems. Accid Anal Prev.

[CR37] Peek-Asa C, Zhang L, Hamann CJ, O'Neal E, Yang J (2023). Direct medical charges of all parties in teen-involved vehicle crashes by culpability. Inj Prev.

[CR38] Ramirez M, Yang JZ, Young T (2013). Implementation evaluation of steering teens safe: engaging parents to deliver a new parent-based teen driving intervention to their teens. Health Educ Behav.

[CR39] Resnicow K, Jackson A, Wang T, Dudley W, Baranowski T (2001). A motivational interviewing intervention to increase fruit and vegetable intake through Black churches: results of the eat for life trial. Am J Public Health.

[CR40] Rubin DB, Schenker N (1991). Multiple imputation in healthcare databases: an overview and some applications. Stat Med.

[CR41] Simons-Morton B (2007). Parent involvement in novice teen driving: rationale, evidence of effects, and potential for enhancing graduated driver licensing effectiveness. J Saf Res.

[CR42] Simons-Morton BG, Bingham CRW, Ouimet MC (2013). The effect of teenage risky driving of feedback from a safety monitoring system: a randomized controlled trial. J Adolesc Health.

[CR43] Summala H, Rajalin S, Radun I (2014). Risky driving and recorded driving offenses: a 24-year follow-up study. Accid Anal Prev.

[CR44] Ver Hoef JM, Voverng P (2007). Quasi-Poisson vs. negative-binomial regression: how should we model overdispersed count data?. Ecology.

[CR45] Watson A, Kaye SA, Fleiter J, Freeman J (2020). Effectiveness of vehicle impoundment for high-range speeding offences in Victoria, Australia. Accid Anal Prev.

[CR46] Williams AF (2003). Teenage drivers: patterns of risk. J Safety Res.

[CR47] Winston FK, Mirman JH, Curry AE (2015). Engagement with the TeenDrivingPlan and diversity of teens’ supervised practice driving: lessons for internet-based learner driver interventions. Inj Prev.

[CR48] Winston FK, Puzino K, Romer D (2016). Precision prevention: time to move beyond universal interventions. Inj Prev.

[CR49] Yang JZ, Campo S, Ramirez M, Krapfl JR, Cheng G, Peek-Asa C (2013). Family communication patterns and teen drivers' attitudes toward driving safety. J Pediatr Health Care.

[CR50] Zakrajsek JS, Shope JT, Greenspan AI (2013). Effectiveness of a brief parent directed teen driver safety intervention (Checkpoints) delivered by driver education instructors. J Adolesc Health.

